# *Euonymus alatus* (Thunb.) Siebold leaf extract enhanced immunostimulatory effects in a cyclophosphamide-induced immunosuppressed rat model

**DOI:** 10.29219/fnr.v67.9422

**Published:** 2023-04-19

**Authors:** Dong Yeop Shin, Byeong Soo Kim, Hak Yong Lee, Young Mi Park, Yong Wan Kim, Min Jung Kim, Hye Jeong Yang, Mi Seong Kim, Jun Sang Bae

**Affiliations:** ^1^Department of Companion and Laboratory Animal Science, Kongju National University, Yesan, South Korea; ^2^INVIVO Co. Ltd., Nonsan, South Korea; ^3^Daegu Cancer Center, Research and Development Unit, DongSung Pharmaceuticals Co. Ltd., Daegu, South Korea; ^4^Korea Food Research Institute, Iseo, South Korea; ^5^Department of Oral Biochemistry, College of Dentistry, Institute of Biomaterial-Implant, Wonkwang University, Iksan, South Korea; ^6^Department of Pathology, College of Korean Medicine, Wonkwang University, Iksan, South Korea

**Keywords:** Euonymus alatus (Thunb.) Siebold, immune enhancement, cyclophosphamide, macrophage, immunosuppressed rat

## Abstract

**Background:**

*Euonymus alatus* (Thunb.) Siebold (EA) is a medicinal plant used in some Asian countries to treat various diseases, including cancer, hyperglycemia, diabetes, urticaria, dysmenorrhea, and arthritis. Owing to the wide range of pharmacological applications of EA, various roles of EA are being studied.

**Objective:**

We evaluated the immune-enhancing effect of EA treatment in a cyclophosphamide (Cy)-induced immunosuppressed rat model.

**Design:**

We analyzed the immune enhancement effect of EA on macrophages by western blotting. In addition, cell viability and natural killer (NK) cell activity were analyzed in splenocytes following EA treatment. For *in vivo* studies, analysis of weekly body weight, spleen weight, immune cell count, cytokine levels, and spleen histological findings was performed following EA administration in Cy-induced immunocompromised rats.

**Results:**

EA significantly increased cell viability and phospho-nuclear factor-kappa B and phospho-extracellular signal-regulated kinase protein levels in the macrophages. EA significantly increased NK cell activity in splenocytes compared with the control group. In Cy-induced immunosuppressed rats, EA administration increased spleen tissue weight and the contents of leukocytes, lymphocytes, granulocytes, intermediate cells, and plasma cytokines (tumor necrosis factor-α and interferon-γ). In addition, improvement in the damaged spleen tissue was observed.

**Conclusions:**

These findings confirm that EA exerts an immune-enhancing effect, thereby suggesting its potential as an immunostimulatory agent or functional food.

## Popular scientific summary

- *Euonymus alatus (Thunb.)* Siebold (EA) is a medicinal plant used in some Asian countries to treat various diseases.
- EA may exerts immuno-enhancement effects in cyclophosphamide-induced immunosuppressed rat.
- EA may be possibly use in developing immunostimulatory agents or functional foods.

Immunity is an important function in maintaining homeostasis in living organisms by inducing biological responses to effectively protect against the invasion of external pathogenic organisms or internal insults. Disorders of the immune system caused by conditions such as immunodeficiency can lead to a variety of diseases, such as infections and cancers (1, 2). Therefore, it is important to identify treatments that improve immune response in immunocompromised individuals. Allopathic medications are typically used to strengthen the immune system. However, these treatments are expensive and often cause side effects (3, 4). To compensate for these limitations, recent studies have identified physiologically active substances that can alleviate the side effects of various drugs on immune function (5, 6).

Natural products with good pharmacological effects, low toxicity, and few side effects on human health have traditionally been widely used in oriental medicine. Among them, *Euonymus alatus* (Thunb.) Siebold (EA) is a medicinal plant predominantly used in China, Korea, and some Asian countries for the treatment of various diseases, such as cancer, inflammation, dysmenorrhea, arthritis, hyperglycemia, and complications of diabetes (7–9). Advances in chemical composition and pharmacological studies on EA have expanded its clinical applications. Moreover, this product is inexpensive, widely available, and has potential for development. Recently, EA has been shown to have various pharmacological activities, including antitumor, anti-inflammatory, antidiabetic, antioxidant, and antibacterial effects (7, 10). Chemicals such as flavonoids, terpenoids, phenylpropanoids, lignans, steroids, and alkaloids have been identified as the major phytochemicals in EA (7, 8). Although various pharmacological effects of EA have been demonstrated in *in vitro* and *in vivo* studies, the effect of EA therapy on immune response remains unclear.

Cyclophosphamide (Cy), an immunosuppressant drug, was used to establish a rat model of chemical-induced immunosuppression (11, 12). Cy has been widely used as an immunosuppressant in organ transplants, autoimmune diseases, and cancer (13, 14). Cy may cause drastic changes in the Th1/Th2 ratio bias, leading to immunosuppression. The immunological effect of Cy decreases lymphocyte proliferation, including the proliferation of T cells, and reduces the production of Th1 cytokines, such as tumor necrosis factor (TNF)-α, interferon (IFN)-γ, interleukin (IL)-2, and IL-12, and Th2 cytokines, such as IL-4, IL-6, and IL-10 (12, 15–17).

Therefore, we investigated whether EA extract exerted immune-enhancing effects in macrophages, splenocytes, and Cy-induced immunocompromised rats.

## Materials and methods

### Samples preparation

The EA was collected in Asan, South Korea, in November 2020, and the plants were air-dried and stored at −20°C before use. Two types of ethanol extracts, EA leaf extracts 1 and 2, were prepared. The dried leaves of EA (2 kg) were extracted with 70% ethanol (2 × 20 L) for 4 h (two times) at 80°C. The extract was filtered through a filter paper. The solvent was evaporated under reduced pressure to yield the concentrated leaf extract 1 (174.0 g). Next, leaf ethanol extract 1 (9.0 g) was suspended in 70% ethanol and fractionated with hexane (four times). A portion of 70% ethanol was evaporated under reduced pressure to yield the concentrated leaf extract 2 (6.7 g). Chlorophyll-free leaf extract 2 was used for *in vitro* and *in vivo* studies. Lipopolysaccharide (LPS) from *Escherichia coli* and Cy or HemoHIM were purchased from Sigma-Aldrich (St. Louis, MO, USA) and Kolmar BNH Co., Ltd. (Sejong, Korea), respectively.

### Cell culture

RAW 264.7 macrophage cells were purchased from the Korean Cell Line Bank (KCLB, Seoul, Korea) and incubated in Dulbecco’s modified Eagle’s medium (DMEM) (Invitrogen, Carlsbad, CA, USA) supplemented with 10% fetal bovine serum (FBS) (Gibco BRL, Gaithersburg, MD, USA) and 1% penicillin/streptomycin (Invitrogen) at 37°C and 5% CO_2_. A single-cell suspension of splenocytes was prepared using tweezers and a 70-μm cell strainer (SPL Life Sciences, Pocheon, Gyeonggi, Korea) after aseptic excision of the spleens of Wistar rats. Cells were washed in Roswell Park Memorial Institute (RPMI)-1640 (Invitrogen) by centrifugation three times (80 × g for 3 min at 4°C). Red blood cell lysing buffer (Sigma-Aldrich) was added for 3 min to remove erythrocytes and then used for the experiment. Isolated splenocytes were cultured in RPMI-1640 medium containing 10% FBS (Gibco BRL) and 1% penicillin and streptomycin (Invitrogen) at 37°C in a humidified incubator with 5% CO_2_.

### Western blot analysis

After dispensing the RAW 264.7 cells (1 × 10^6^ cells/mL) in a 100 mm dish, the cells were treated with EA (0, 5, 10, and 50 µg/mL) or LPS (300 ng/mL) for 24 h. The cells were washed using phosphate buffered saline (PBS) and lysed using PRO-PREP^TM^ protein extraction solution (iNtRON Biotechnology, Korea). The lysates were centrifuged at 14,000 rpm for 10 min at 4°C, and the protein concentration was quantified using Bradford reagent (Bio-Rad, Hercules, CA, USA). Proteins were separated by sodium dodecyl sulfate polyacrylamide gel electrophoresis (SDS-PAGE) and transferred onto polyvinylidene difluoride (PVDF) membranes. The membrane was blocked with 5% skim milk solution for 1 h. The membranes were incubated with primary antibodies at 4°C. The primary antibodies used for western blotting were as follows: NF-kB p65 (Cell Signaling Technology, Beverly, MA, USA), phospho-NF-κB p65 (Cell Signaling), p44/42 mitogen-activated protein kinase (MAPK) (Erk1/2) (Cell Signaling), phospho-p44/42 MAPK (Erk1/2) (Thr202/Tyr204) (Cell Signaling), and β-actin (Sigma-Aldrich). After washing the membranes with Tris-buffered saline containing Tween 20 (TBS-T) and then treated with secondary antibodies containing horseradish peroxidase (HRP) for 1 h, the immunoreactive bands were treated with enhanced chemiluminescence (ECL) solution (EZ-Western Lumi Pico, DoGen, Korea) and detected by a C-Digit western scanner (LI-COR, Lincoln, NE, USA).

### Cell viability assay

RAW 264.7 cells (1 × 10^4^ cells/90 μL/well) and the isolated splenocytes (1 × 10^6^ cells/90 μL/well) were seeded in 96-well plates, treated with dose-dependent of EA, and incubated for 24 h at 37°C. After 24 h, 10 μL of water soluble tetrazolium salt (WST)-1 solution (ITSBio, Inc., Seoul, Korea) was added and incubated for 1 h. The optical density was measured at 405 nm using a multi-detection reader (Infinite 200, TECAN Group Ltd, Switzerland).

### Natural killer cell activity assay

Natural killer (NK) cell activity was assessed as described previously (17). Briefly, AR42J rat pancreatic tumor cells were purchased from the American Type Culture Collection (ATCC, Manassas, VA, USA). AR42J cells were used as target cells for the NK cell activity assay and control, or EA-treated splenocytes were used as effector cells. Splenocytes were cocultured with AR42J cells in 96-well plates at a ratio of effector cells to target cells (20:1) and incubated for 24 h. The viability of AR42J cells was measured by the CytoTox detection kit (TaKaRa, Shiga, Japan). NK cell activity was calculated as viability of AR42J cells compared to control cells.

### Animals and experimental design

Specific pathogen-free (SPF) 5-week-old male Wistar rats (*n* = 60) were purchased from Orient Bio Inc. (Seongnam, Gyeonggi-do, Korea) and acclimatized to the following conditions for 7 days: 12-h light/dark cycle; temperature, 23 ± 1°C; humidity, 50 ± 5%; illumination, 150–300 lx. During the adaptation period, the standard feed and filtered drinking water were changed daily to allow free intake. After the acclimatization period, the experimental animals (*n* = 60) were separated using the measured body weight values. The mean values between the groups were equally divided into six groups of 10 animals each: normal control group (normal), immunosuppression group (control), immunosuppression + EA 50 mg/kg/day group (EA 50), immunosuppression + EA 100 mg/kg/day group (EA 100), immunosuppression + EA 300 mg/kg/day group (EA 300), and immunosuppression + HemoHIM 1,000 mg/kg/day group (HemoHIM 1,000). An immunosuppression induction model was established by oral administration of Cy (5 mg/kg). HemoHIM (1,000 mg/kg) was used as a positive control. The concentrations of Cy and HemoHIM used in the experiments have been described previously (17). All drugs and vehicles were administered via oral gavage using an oral zonde for 4 weeks. Body weight was measured once a week at a certain time. All animal experiments were approved by the Institutional Animal Care and Use Committee of INVIVO Co. Ltd. (approval number IV-RB-17-2105-09-01).

### Complete blood cell count and cytokine analyses

These analyses were performed as previously described (17). Briefly, after the final administration of all drugs and vehicles, whole blood was collected after inhalation anesthesia (isoflurane, USP) and divided into ethylenediaminetetraacetic acid (EDTA)-coated tubes (Becton Dickinson Caribe, Ltd.) and conical tubes for analysis. The numbers of total white blood cells (WBCs), lymphocytes, granulocytes, and mid-range absolute counts (Mid) collected in EDTA-coated tubes were analyzed using a Hemavet 950 counter (Drew Scientific Group, Dallas, TX, USA). For cytokine analysis, blood collected in a conical tube was coagulated at room temperature for 30 min and then separated by centrifugation at 3,000 rpm for 10 min to collect serum. The separated serum was analyzed for TNF-α, IFN-γ, and IL-6 levels using an ELISA kit (R&D Systems, Minneapolis, MN, USA).

### Histological analysis

After euthanizing the animals, the extracted spleen tissues were weighed, re-fixed by trimming the samples fixed in 10% formalin solution, then embedded in paraffin, and sectioned to a thickness of 4 μm. For hematoxylin–eosin staining, paraffin was removed from xylene, dehydrated, and stained with hematoxylin and eosin for 4 and 2 min, respectively. Thereafter, sections were examined using a microscope (BX50 F4; Olympus, Fukuoka, Japan).

### Statistical analysis

All data are presented as mean ± SEM using a statistical software (SPSS ver.12.0, SPSS Inc., Chicago, IL, USA). One-way analysis of variance (ANOVA) and Duncan’s test were performed for statistical analysis according to the statistical significance test for each experimental group. Differences between groups were considered significant when *p* < 0.05.

## Results

### Effect of EA-mediated cell viability in RAW 264.7 cells

**Fig. 1:**
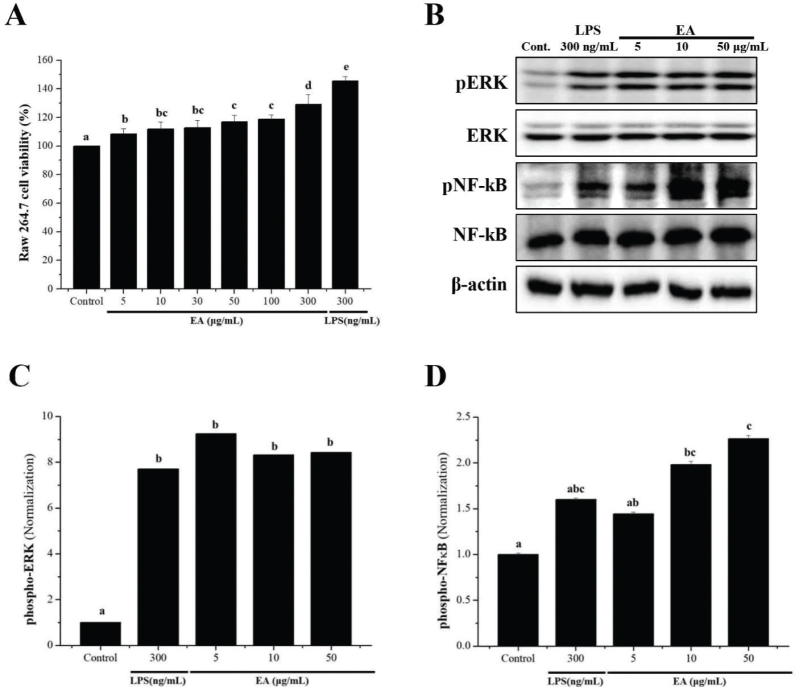
Effects of EA extract on cell viability and levels of phospho-NF-κB and phospho-ERK in RAW 264.7 cells. (A) RAW 264.7 cells (1 × 10^4^ cells/90 μl/well) were seeded in 96-well plates, treated with EA (0, 5, 10, 30, 50, 100, and 300 μg/mL) or LPS (300 ng/mL), and incubated at 37°C and 5% CO_2_ for 24 h. After 24 h, cell viability was measured using the WST-1 assay. (B) RAW 264.7 cells (1 × 10^6^ cells/mL) were aliquoted in a 100 mm dish and treated with EA (0, 5, 10, and 50 µg/mL) or LPS (300 ng/mL) for 24 h. The expression levels of phospho-ERK and phospho-NF-κB were analyzed by western blotting using anti-NF-κB p65, p-NF-κB p65, p44/42 MAPK (Erk1/2), and p-p44/42 MAPK (Erk1/2) antibodies. (C, D) Western blots were quantified using the Quantity One 4.6.6 software. Bars labeled with different superscripts indicate significant differences (*p* < 0.05, vs. control). The results are expressed as mean ± SEM of at least three independent experiments (*n* = 3).

We first used a cell viability assay to confirm the cytotoxicity of macrophages in a dose-dependent manner before examining the immune-boosting effects of the EA extract on macrophages. These results showed that the cell viability of RAW 264.7 increased after treatment with EA (0, 5, 10, 30, 50, 100, and 300 μg/mL) in a dose-dependent manner. In addition, the viability of RAW 264.7 cells was increased even in the LPS-treated group (300 ng/mL) used as a positive control (Fig. 1A).

### Phosphorylation of ERK and NF-*κ*B proteins by EA in RAW 264.7 cells

To investigate the immunostimulatory effect of EA, western blotting was performed to analyze the expression and phosphorylation of extracellular signal-regulated kinase (ERK) and nuclear factor-kappa B (NF-κB) proteins in RAW 264.7 cells. Our results showed that the phosphorylation of ERK and NF-κB increased after treatment with EA (0, 5, 10, and 50 μg/mL). Interestingly, p-NF-κB levels were higher in the EA 10 and 50 μM treated groups than in the LPS-treated group (300 ng/mL) (Fig. 1B). Each protein bands was normalized, and the relative expression levels in the control group were analyzed (Fig. 1C and D).

### Effects of EA on cell viability and NK cell activity in splenocytes

**Fig. 2:**
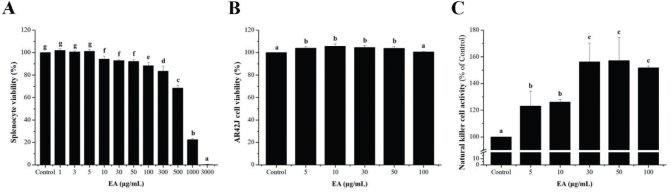
Effects of EA on NK cell activity in splenocytes. (A) Isolated splenocytes (1 × 10^6^ cells/90 μl/well) were seeded in 96-well plates and treated with EA (1–3,000 μg/mL) for 24 h. Cell viability was determined using a WST-1 assay. (B) Splenocytes were cocultured with AR42J cells (target cells) in 96-well plates at a ratio effector cells to target cells (20:1), followed by treatment with EA (0, 5, 10, 30, 50, and 100 μg/mL) and incubated for 24 h in a 5% CO_2_ incubator. (C) NK cell activity was calculated as the survival rate of AR42J cells compared to that of control cells. Bars labeled with different superscript numerals indicate *p* values < 0.05. Values are presented as mean ± SEM of three independent experiments (*n* = 3).

To determine the toxic concentration of EA in splenocytes, the cells were treated with various EA concentrations (1–3,000 μg/mL). After 24 h, cell viability was unaffected at EA concentrations of <100 μg/mL. At a concentration of 300 μg/mL or higher, it was confirmed that the survival rate decreased to 90% or less (Fig. 2A). Based on these results, up to 100 μg/mL concentration, which did not show cytotoxicity, was set as the highest concentration of EA, and then the NK cell activity was analyzed. We performed an NK cell activity assay to evaluate the effect of the EA extract on non-specific cell-mediated immunity. We first confirmed the viability of AR42J cells treated with EA extract. At a concentration of <50 μg/mL, the survival rate was slightly higher than that of the control group, and at a concentration of 100 μg/mL, cell viability was similar to that of the control group (Fig. 2B). As shown in Fig. 2C, NK cell activity significantly increased after treatment with EA compared with that in the control group.

### Effects of EA on body and spleen weight loss in Cy-induced rats

**Fig. 3:**
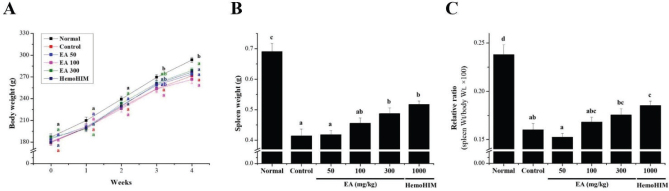
Effect of EA on body and spleen weight loss in Cy-induced rats. Male Wistar rats (*n* = 60) were divided into six groups of 10 animals each and administered orally for 4 weeks: normal control group (normal), Cy-treated (5 mg/kg/day) group (control), Cy + EA (50 mg/kg/day) group (EA 50), Cy + EA (100 mg/kg/day) group (EA 100), Cy + EA (300 mg/kg/day) group (EA 300), and Cy + HemoHIM (1,000 mg/kg/day) group (HemoHIM 1,000). (A) Body weight was measured once a week to confirm the change in body weight over 4 weeks. Indices of (B) spleen and (C) relative ratio (weight/body weight × 100). Bars labeled with different superscript numerals indicate *p* values < 0.05. Data are expressed as mean ± SEM (*n* = 10)*.*

To confirm the effect of the EA extract on the immunocompromised rat model using Cy, the weekly body weight change between each group was verified. At the end of the experiment, the 4th week, the control (only Cy-treated) group significantly lower at 271.59 ± 4.70 g compared to 293.46 ± 3.44 g in the normal group. On the other hand, the body weight of the experimental group EA 50, EA 100, and EA 300 orally administered with the Cy and the EA extract (50, 100, and 300 mg/kg) was 273.87 ± 2.44 g, 266.71 ± 5.75 g, and 278.89 ± 3.59 g, respectively. The body weight of the positive control group (HemoHIM 1,000 mg/kg) was 276.74 ± 4.23 g. There was a significant difference between the control and experimental groups compared with the normal group (Fig. 3A). In addition, spleen tissues were weighed in immunocompromised rats. The spleen index, a representative immune organ, was significantly decreased by oral administration of Cy. However, these reductions in Cy-treated rats were significantly recovered by the oral administration of EA in a dose-dependent manner. The spleen tissue weights of each group were as follows: normal group: 0.69 ± 0.03 g; control group: 0.41 ± 0.02 g; EA 50 group: 0.42 ± 0.01 g; EA 100 group: 0.46 ± 0.02 g; EA 300 group: 0.49 ± 0.02 g; and HemoHIM 1,000 group: 0.52 ± 0.01 g (Fig. 3B). These results were similar in terms of the relative proportions of body weight (Fig. 3C).

### Effects of EA on spleen damage in Cy-induced immunosuppressed rats

**Fig. 4:**
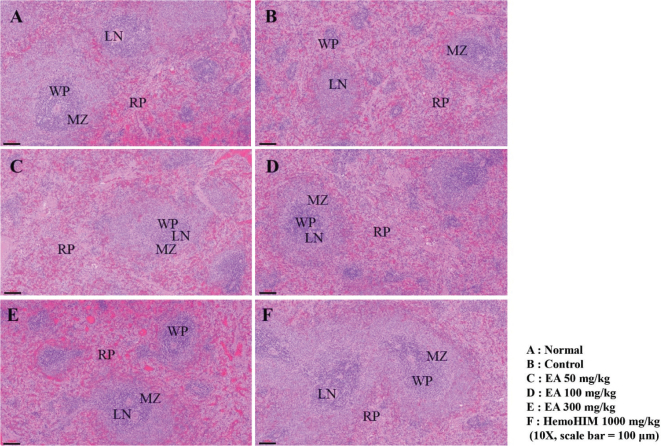
Effects of EA on spleen damage in immunosuppressed rats. Wistar rats were orally administered saline, Cy (5 mg/kg/day), Cy + EA (50, 100, and 300 mg/kg/day), or HemoHIM (1,000 mg/kg/day) daily for 4 weeks. Subsequently, damage to the spleen was histologically analyzed. Representative images of sectioned (A) normal (saline-treated) group, (B) control (only Cy-treated) group, (C–E) Cy + EA-treated groups; (C) EA 50, (D) EA 100, (E) EA 300, or (F) Cy + HemoHIM-treated group (HemoHIM 1,000). Scale bar = 100 μm. CV, central vein; LN, lymph nodule; MZ, marginal zone; RP, red pulp; WP, white pulp.

In this study, splenic tissue lesions in each group were observed using an optical microscope to determine the effect of the EA extract on splenic morphological changes following Cy treatment. As a result of observing the spleen tissue lesions under a microscope, it was observed that the normal group had a white pulp surrounding the central artery, and the lymph nodes were at the edges, so that the border with the red pulp was clear (Fig. 4A). In contrast, in the control group treated with Cy only, atrophy of the white pulp in the spleen and lymphoid depletion were observed, confirming immune suppression by Cy (Fig. 4B). However, in the EA 50 group, the marginal zone (MZ), the zone that distinguishes the white pulp from the red pulp, was not clear, but the atrophy of the white pulp, which was severely observed in the control group, improved (Fig. 4C). In the EA 100 group, the degree of atrophy of the white pulp of the spleen was lower, and the damage was relatively reduced (Fig. 4D). These patterns were conspicuous in the EA 300 group, where the marginal area around the red pulp was clearly visible, and there was no collapse of the white pulp (Fig. 4E). In the case of the HemoHIM 1,000 group (positive control), tissue disruption and condensation did not appear, and it was significantly improved compared with the control group (Fig. 4F).

### Effects of EA on immune cell number in Cy-induced immunosuppressed rats

**Fig. 5:**
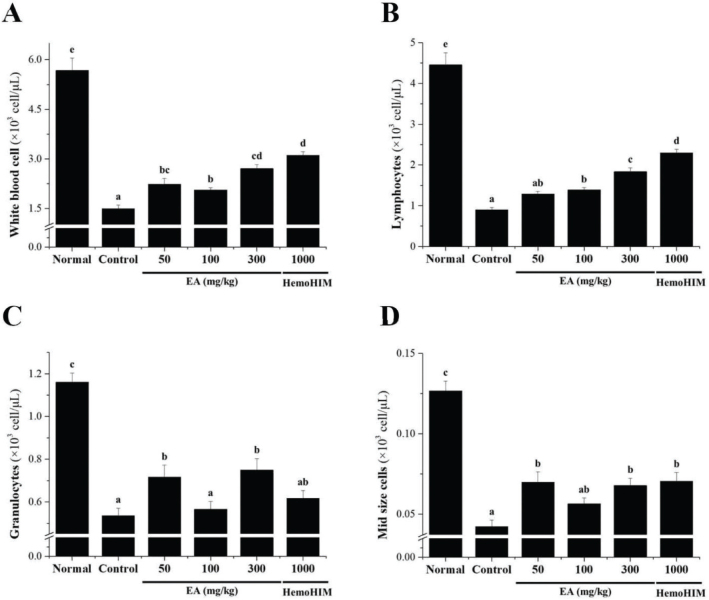
Effects of EA on immune cell number in Cy-treated rats. Wistar rats were orally administered saline (normal), Cy (5 mg/kg/day) (control), Cy + EA (50, 100, and 300 mg/kg/day) (EA 50, EA 100, and EA 300, respectively), or HemoHIM (1,000 mg/kg/day) (HemoHIM 1,000) daily for 4 weeks. A complete blood cell count (CBC) test was performed using a blood analyzer. The numbers of (A) total WBCs, (B) lymphocytes, (C) granulocytes, and (D) mid-sized cells in blood samples were determined using a Hemavet 950 system. Bars labeled with different superscript numerals indicate *p* values < 0.05. Data are expressed as mean ± SEM (*n* = 10).

Hematological analysis was carried out to determine the effect of the EA extract on the level of immune cells in the blood of immunosuppressed rats. In the Cy-induced immunosuppression rat model, the number of immune cells, such as total WBCs, lymphocytes, granulocytes, and mid-size cells (Mid), was significantly reduced compared with that in the normal group. However, the number of immune cells increased after EA-administered (Fig. 5).

### Effect of EA on the cytokine level of serum in Cy-induced immunosuppressed rats

**Fig. 6:**
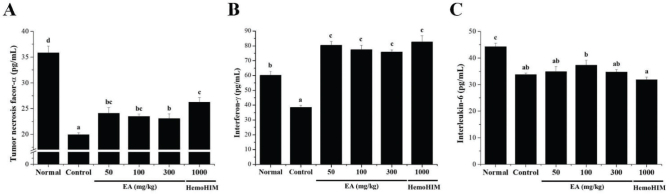
Effects of EA on serum cytokine levels in rats with Cy-induced immunosuppression. Wistar rats were orally administered saline (normal), Cy (5 mg/kg/day) (control), Cy + EA (50, 100, and 300 mg/kg/day) (EA 50, EA 100, and EA 300, respectively), or HemoHIM (1,000 mg/kg/day) (HemoHIM 1,000) daily for 4 weeks. The separated serum was analyzed for (A) TNF-α, (B) IFN-γ, and (C) IL-6 levels, using an ELISA kit. Bars labeled with different superscripts indicate significantly different values (*p* < 0.05). Data are presented as mean ± SEM (*n* = 10).

To confirm the effect of EA on the serum cytokine content in immunocompromised rats by Cy-administration, the amounts of TNF-α, IFN-γ, and IL-6 were analyzed 4 weeks after sample administration. In the Cy-administered group, TNF-α, IFN-γ, and IL-6 levels were significantly lower than those in the normal group. Interestingly, decreased TNF-α and IFN-γ levels in the control group increased when EA (50, 100, and 300 mg/kg) and HemoHIM (1,000 mg/kg) were administered (Fig. 6A and B). However, IL-6 levels were not different from those in the control group, despite EA administration (Fig. 6C).

## Discussion

Drugs derived from natural products have the advantage of low toxicity than chemical drugs and have long been the basis for the treatment of human diseases (5, 6, 18). Therefore, various natural product-based studies have been conducted in major therapeutic areas, such as cancer, immune mechanisms, anti-infection, diabetes, and metabolic disorders (2, 18). Among these candidates, EA extracts have been extensively studied *in vitro* and *in vivo* (7, 19–21), but the effect of EA treatment on the immune response remains unclear. Therefore, in this study, the immunostimulatory effects of EA were investigated in macrophages, splenocytes, and an immunosuppressed rat model.

Our results showed that EA extract increased the viability of the RAW 264.7 cells and increased levels of phospho-NF-κB and phospho-ERK proteins. In addition, there was a significant increase in NK cell activity after EA exposure. In an *in vivo* study, when EA extract was administered to immunosuppressed rats treated with Cy, the weight of the spleen, a representative immune organ, was significantly increased. In addition, oral administration of EA increased the number of immune-related serum cytokines (TNF-α and IFN-γ) as well as immune cells (total WBC, lymphocytes, granulocytes, and Mid) and restored normal splenic histology. These findings suggest that EA restored the Cy-induced immunosuppressive response.

Leukocytes in the spleen are composed of various cells, such as T cells, B cells, dendritic cells, and macrophages with different immune functions. The proliferation of macrophages and lymphocytes is important in the early activation phases of cellular and humoral immune responses (22, 23). Macrophages not only are involved in the initiation of the mononuclear phagocyte system but also have pro-inflammatory and anti-inflammatory properties at the same time, and their functional flexibility is determined by the immunological microenvironment (24). Macrophages are known to be activated by the Th1 cytokine IFN-γ and alternatively activated by Th2 cytokines IL-4 and IL-13. In M1 macrophages, genes related to inflammation are overexpressed due to an increase in inflammatory cytokine expression due to macrophage galactose N-acetyl-galactosamine-specific lectin 1 (MGL1) deletion and upregulation of the dendritic cell marker CD11c, whereas M2 macrophages are responsible for the termination of inflammatory reactions or tissue regeneration (25–27). Stimulation of immune cells, such as macrophage survival, may consequently promote the secretion of cytokines, potentially explaining their immune-enhancing and anticancer abilities (27). In this study, the EA extract significantly increased the macrophage viability rate in a dose-dependent manner compared to the control group (untreated group). These findings suggest that EA promotes non-toxic and immune-enhancing effects in macrophages. The activation of LPS-induced macrophages produces pro-inflammatory cytokines, such as IL-1, TNF-α, IL-6, and IFN-γ and induces MAPK-dependent phosphorylation of p38, c-Jun NH2-terminal kinase (JNK), and ERK (28–30). Additionally, NF-κB is a major activator for the TNF-α production in macrophages and is known to play a major role in controlling the expression of proteins involved in immune, inflammatory, and acute-phase responses (30–32). MAPK and NF-κB signaling play critical roles in the immune response (28–32). A previous study reported that several natural products could show immune enhancement by activating the phosphorylation of inducible Nitric Oxide Synthase (iNOS), cyclooxygenase-2 (COX-2), NF-κB, and MAPKs (JNK, ERK, and p38) in RAW-264.7 cells (17, 33–35). Our results showed that p-ERK and p-NF-κB protein levels in the EA-treated group were higher than in the control group. Previous studies have reported that EA increases NO and the TNF-α production via NF-κB activation (36). These results are thought to be the result of EA playing a positive role in activating the cytokine of Th1 to stimulate immune factors in the body and increase NK cell activity (37). Taken together, these findings suggest that EA induces immunostimulatory effects through the regulation of ERK and NF-κB signaling in macrophages.

NK cells play an important role in the initial immune response and can target and kill virus-infected cells and cancer cells. It is also activated by the stimulation of cytokines and chemokines and is one of the methods that can lead to the activation of innate and adaptive immune cells (38, 39). Our results showed that the EA-treated group had significantly increased NK cell activity. According to a previous report, NK cells are activated by IFN- or macrophage-derived cytokines, and by recognizing changes in major histocompatibility complex (MHC) class expression and blocking the activation of non-infected cells, only infected cells selectively induce apoptosis (39). In this result, EA may play an effective role in activating NK cells to clear cells infected with viruses and bacteria and may enhance cell-mediated immune responses.

Cy administration induces physiological phenomena, such as bone marrow failure, anemia, thrombocytopenia, and growth retardation and damages the spleen and thymus, which are representative immune organs, leading to a decrease in immune function in the body (13, 14). Several studies have reported that Cy-induced immunosuppression *in vitro* and *in vivo* significantly reduces spleen and thymus weight, and blood cell count, damages splenic tissue, and inhibits splenic NK cell and Tc cell activity (15–17, 40). In this study, the spleen weight was reduced by Cy treatment, and the index restored by EA treatment. In particular, in the high-dose EA-treated group, the spleen weight recovered close to that of the HemoHIM group, which was the positive control group. Furthermore, as a result of observing spleen tissue lesions in each experimental group according to Cy treatment, the collapse of the white pulp and cell condensation in the red pulp observed in the control group showed a tendency to gradually improve in the EA extract-administered group. In particular, in the experimental group, in which the EA extract was administered at a high concentration, the white pulp was evenly distributed around the central vein, and the borders of the marginal regions were clearly separated, which significantly reduced the spleen tissue damage caused by Cy. These results show that the splenic tissue damage caused by immunosuppressive substances can be repaired by EA treatment. In addition, WBCs such as T and B cells, monocytes, and macrophages play an important role in cellular immunity and resistance to infectious diseases. The function of WBCs can be enhanced by natural immune stimulants, as reported previously (41, 42). Inflammatory M1 macrophages are also found in chronically inflamed tissues and have been shown to activate other immune cells such as monocytes, neutrophils, and T cells (43). In previous studies, the contents of total leukocytes, lymphocytes, granulocytes, and mid-size cells (Mid) were significantly decreased in the Cy-treated group compared with those in the normal group (12, 15, 17, 40). As in the previous study, our results showed that the decreased number of immune cells in Cy-induced immunosuppressed rats significantly increased the number of total leukocytes, lymphocytes, granulocytes, and Mid after administration of EA extract. These results show that EA extract can activate immunostimulatory and reduce Cy toxicity by acting on blood cells that perform immune functions in the body. Collectively, these findings suggest that EA exerts an immune-enhancing effect by protecting against Cy-induced immune cell and tissue damage.

In the early stages of infection, macrophages release various cytokines and chemokines to attract NK cells to the infection site (44). In previous studies, it was reported that the low survival rate of TNF-α-deficient mice was due to spleen damage, and that deficiency in IFN-γ results in a more severe disease in response to a variety of pathogens (45, 46). In addition, IL-6-deficient mice showed reduced phagocytic activity of macrophages during infection (47). This innate immune response activates other inflammatory cells to enhance the adaptive immune response (44). As essential regulatory cells of the adaptive immune system, T cells promote the secretion of IL-2, TNF-α, and IFN-γ from Th1 cells and IL-4, IL-6, and IL-10 from Th2 cells (15–17, 30). TNF-α produced by lymphocytes and macrophages protects the host from infectious pathogens (45). In a previous study, EA increased NO and TNF-α production through NF-κB activation (36). Our results suggest that the EA extract may be involved in the production of macrophage-related cytokines TNF-α and IFN-γ through p-ERK and p-NF-κB signaling in RAW 264.7 macrophages. Recognition of LPS by Toll-like receptor 4 in macrophages is known to increase the production of pro-inflammatory cytokines, such as TNF-α, IL-1β, and IL-6, through activation of the NF-κB signaling pathway (48). Previous studies have reported that Cy-induced immunosuppression *in vitro* and *in vivo* significantly reduces the levels of cytokines, such as TNF-α, IFN-γ, IL-2, IL-6, and IL-10 (12, 15–17). Our results confirmed that the decreased levels of TNF-α and IFN-γ in Cy-induced immunosuppressed rats significantly increased when EA was administered. However, contrary to expectations, the IL-6 levels did not change. These results show that EA promotes the secretion of TNF-α and IFN-γ by regulating Th1 cells among the T cell subtypes. Taken together, these findings suggest that EA may improve immune function by increasing the levels of immunostimulatory cytokines. However, further studies are necessary to more accurately determine the immunostimulatory effects of EA.

## Conclusions

In conclusion, EA extract increased cell viability and the phosphorylation levels of NF-κB and ERK proteins in macrophages and also increased the activity of NK cells in splenocytes. In *in vivo* studies, EA extract increased spleen tissue weight and restored the spleen damaged by Cy. In addition, it was found to be effective in increasing immunity by increasing the content of immune cells and levels of TNF-α and IFN-γ in the blood. Therefore, these results suggest that EA extract is effective in enhancing immunity and may be helpful in the development of immunostimulatory agents or functional products.

## Data Availability

The data used to support the findings of this study are included within the article.
